# The Impact of Social Influence on the Intention to Use Physician Rating Websites: Moderated Mediation Analysis Using a Mixed Methods Approach

**DOI:** 10.2196/37505

**Published:** 2022-11-14

**Authors:** Bernhard Guetz, Sonja Bidmon

**Affiliations:** 1 Department of Marketing and International Management Alpen-Adria-Universitaet Klagenfurt Klagenfurt am Woerthersee Austria

**Keywords:** social influence, eHealth literacy, patient satisfaction, physician rating websites

## Abstract

**Background:**

Physician rating websites (PRWs) have become increasingly important in the cross-section between health and digitalization. Social influence plays a crucial role in human behavior in many domains of life, as can be demonstrated by the increase in high-profile influential individuals such as social media influencers (SMIs). Particularly in the health-specific environment, the opinion of family and friends has a significant influence on health-related decisions. However, so far, there has been little discussion about the role of social influence as an antecedent of behavioral intention to use PRWs.

**Objective:**

On the basis of theories of social psychology and technology acceptance and theories from the economic perspective, this study aimed to evaluate the impact of social influence on the behavioral intention to use PRWs.

**Methods:**

We conducted 2 studies by applying a mixed methods approach including a total of 712 participants from the Austrian population. The impact of social influence on the behavioral intention to use PRWs was investigated through linear regression and mediation and moderated mediation analysis using the PROCESS macro 4.0 in SPSS 27 (IBM Corp).

**Results:**

The 2 studies show similar results. In study 1, an experiment, no direct effect of social influence on the behavioral intention to use PRWs could be detected. However, an indirect effect of social influence on the behavioral intention to use PRWs via credibility (*b*=0.572; *P*=.005) and performance expectancy (*b*=0.340; *P*<.001) could be confirmed. The results of study 2, a cross-sectional study, demonstrate that social influence seems to have a direct impact on the behavioral intention to use PRWs (*b*=0.410; *P*<.001). However, when calculating the proposed mediation model, it becomes clear that this impact may partly be explained through the 2 mediator variables—credibility (*b*=0.208; *P*<.001) and performance expectancy (*b*=0.312; *P*<.001). In contrast to the observed direct and indirect effect, neither demographic nor psychographic variables have a significant moderating impact on the influencing chain in study 2.

**Conclusions:**

This study provides an indication that social influence has at least an indirect impact on the behavioral intention to use PRWs. It was observed that this impact is exerted through credibility and performance expectancy. According to the findings of both studies, social influence has the potential to boost the use of PRWs. As a result, these web-based networks might be a promising future interface between health care and digitalization, allowing health care practitioners to gain a beneficial external impact while also learning from feedback. Social influence nowadays is not just limited to friends and family but can also be exerted by SMIs in the domain of PRW use. Thus, from a marketing perspective, PRW providers could think of collaborating with SMIs, and our results could contribute to stimulating discussion in this vein.

## Introduction

### Background

The number of websites where patients can publicly share their health care experiences has grown rapidly in recent years [1]. These web-based platforms are characterized by patients sharing their subjective perceived health experience with the entire web-based community by creating qualitative reviews and quantitative ratings [2]. In addition, there is an increasing number of patients who use these websites to make their health-related decisions as well as to search for and select a suitable health service provider [3]. Especially for health care providers, those web-based portals represent a cost-efficient possibility to achieve a positive external impact [4]. This shows that physician rating websites (PRWs) provide the opportunity to evaluate incidents in the health sector and make evidence-based decisions by referring to existing evaluations on rating sites [5].

### Theoretical Background

We built our conceptual framework on insights from several theoretical domains. These can be categorized as theories from the social psychological perspective, theories of technology acceptance, and theories from the economic perspective. From a social psychological perspective, the theory of reasoned action (TRA) [6] and its extension, the theory of planned behavior (TPB), can be used as a framework for this study [7]. According to the TRA, it is assumed that individuals’ *attitudes* and *subjective norms* shape their *behavioral intentions* as well as their *behavior* [8]. It is proposed that individuals are more likely to perform a specific *behavior* if they have a positive *attitude* toward this behavior and believe that others want them to perform it (*subjective norm*) [9]. The TRA has formed the theoretical underpinning of many empirical studies so far. As the meta-analysis by Sheppard et al [10] in 1988 could demonstrate, the empirical results of several studies contribute to support the TRA [10]. However, the proposed influencing chain was further extended through the more sophisticated version, the TPB [11]. According to this theory, there are 3 independent core components that shape an individual’s *behavioral intentions* [12]. These include *attitudes* and *subjective norms* but also *perceived behavioral control* [13]. In this context, again, *attitude* describes the individual’s view of a particular behavior, and *subjective norms* describe what others might think about the particular behavior [14]. However, *perceived behavioral control* is an individual’s sense of control over their own behavior and represents an exogenous variable that, in contrast to the other 2, affects both *behavioral intention* and *behavior* itself [7].

The second stream of theories that provide both the foundation and framework for this study are the theories of technology acceptance and technology use. These theories are based on the TPB and integrate further theories, factors, and modifications depending on their individual characteristics [15-18]. The pioneering theory of this kind was the technology acceptance model (TAM), in which it was assumed that *perceived usefulness* and *perceived ease of use* influence the attitude toward using [19,20]. According to this model, *attitude toward using* represents the decisive predictor for *actual system use* [21]. Further developments of the original TAM were published as TAM 2 [22] and TAM 3 [23]. The currently prevailing theory of technology acceptance was developed on this basis. The so-called Unified Theory of Acceptance and Use of Technology (UTAUT) [24,25] and its further development, the Extended UTAUT [26], propose factor models that are characterized by a vast number of independent influencing variables [27]. These include, for example, *performance expectancy* or *social influence* [28]. According to the UTAUT, these factors affect the *use behavior* regarding new or adapted technologies whereby the impact can be obtained directly or indirectly via *behavioral intention* [29].

Finally, from an economical perspective, the theory of information economics (TIE) [30] and the concept of source similarity [31] also contribute to the theoretical underpinning of this study. According to the TIE, consumers are not able to sufficiently assess the quality of credence products [30]. In the medical context, this means that patients are dependent on additional sources beyond their mere subjective perception to be able to assess the quality of the medical encounter and the medical treatment involved [32,33]. The concept of similarity, in which it is assumed that the recipient of a piece of advice evaluates the quality of the source, could be valuable in this context [34]. According to this theoretical concept, the decision for or against advice received is dependent on the transmitter’s attributed expertise and similarity to the perceiver’s point of view [35]. Sources that are perceived as similar seem to have a significant impact from the perceiver’s perspective because of the ascription of similar needs and expectations [31]. These assumptions show that advice from a person with a high similarity might lead to a change in behavior [36] and, thus, inter alia could also contribute to an increase in the *behavioral intention* to use PRWs.

The effect of *social influence* on the *behavioral intention* to use a new or adapted technology is well known. For example, previous studies have already shown that *social influence* has an impact on the *behavioral intention* to use mobile-based assessments [37], Instagram messaging [38], web-based banking [39], eHealth services [40], social media [41], e-government [42], e-learning [43], and accounting platforms [44]. Although this review is not exhaustive and includes only a fraction of the studies that have investigated the relationship between *social influence* and the behavioral use intention, to the best of our knowledge, the relationship between *social influence* and *behavioral intention* to use PRWs has not been investigated so far. For this reason, we conducted 2 studies by applying a mixed methods approach to investigate the direct and indirect effects of *social influence* on the *behavioral intention* to use PRWs, including potential moderation effects. The following section elaborates on this.

### Determinants of Behavioral Intention to Use PRWs

The *behavioral intention* to use a new or adapted technology refers to the strength of the ambition to perform a particular action [6,22]. On the basis of various studies in this field (eg, Krueger and Carsrud [45], Tonglet et al [46], Hardeman et al [47], Anderson and Schwager [48], Hoogenbosch et al [49], and Venkatesh and Zhang [24]), it can be assumed that the *behavioral intention* to use a new technology plays a crucial role in forecasting individuals’ actual or future use behavior.

#### Social Influence

Consumer decisions in the product or service sector are often strongly influenced by individuals who have an impact on the customers’ behavior [50]. The whole boom of influencer marketing nowadays is more or less based on this fact. However, in the medical field, the degree of perceived uncertainty from the patients’ perspective is frequently very high, which leads to the fact that *social influence* has a very strong effect on patients’ decision-making behavior [51]. Moreover, studies have shown that individuals whom patients feel closely related to have a strong impact on a variety of health-related decisions such as the choice of physician, the therapeutic method, or the frequency of medical consultation [52,53]. Beyond that, *social influence* was represented as subjective norm and was already an integral part of the TPB [11] and of subsequent theories from the field of technology acceptance [25]. For this reason, we focused on *social influence* as the independent variable and propose the following hypothesis: a positive *social influence* leads to a higher *behavioral intention* to use PRWs (hypothesis 1).

**Figure 1:**
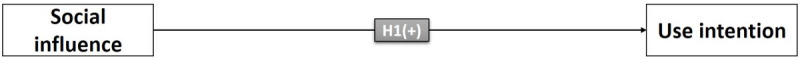
Direct effect of social influence on the behavioral intention to use physician rating websites. H: hypothesis.

Figure 1 shows the proposed direct effect of *social influence* on the *behavioral intention* to use PRWs.

In hypotheses 2 to 6, we describe indirect effects, which increase the *behavioral intention* to use PRWs through *credibility* and *performance expectancy* based on *social influence*. Owing to this effect in which *credibility* and *performance expectancy* act as mediator variables, a potential direct effect of *social influence* on the *behavioral intention* to use PRWs should be weakened [54]. This assumption is based on the fact that mediator variables generally affect the direct effect of the independent variable on the dependent variable [55]. As we assume that the impact of *social influence* on the *behavioral intention* to use PRWs can partly be explained through *credibility* and *performance expectancy*, we argue that, in the mediation model, the direct effect between *social influence* and the *behavioral intention* to use PRWs gets weaker compared with the single linear regression (hypothesis 1a).

#### Credibility

The concept of *credibility* describes the level of believability of a transmitter judged by the information perceiver [56]. Perceived *credibility* has an important impact on the whole consumer decision-making process, especially in the case of decisions under conditions of uncertainty [57]. In this context, it was shown that *social influence* not only can change or reinforce the attitude toward new or unknown subjects but also affects subject attributes such as the *credibility* of an information source [58]. Thus, *credibility* is a construct that is strongly controlled by *social influence* [59]. This leads to our second hypothesis: a more positive *social influence* leads to a higher *credibility* of PRWs (hypothesis 2).

In addition to *social influence*, the impact of credibility on behavioral use intention has also been investigated in the past [60]. In the context of technology acceptance, it was shown that credibility exerts a direct impact on the *behavioral intention* to use a new or adapted technology [61]. For this reason, we propose the following: a higher *credibility* leads to a higher *behavioral intention* to use PRWs (hypothesis 3).

#### Performance Expectancy

With regard to use of new technologies, *performance expectancy* is based on the fact that the use of new systems and the associated change of behavior can lead to an improvement of the current state [25]. This means that the desire and motivation to use and accept a new application or technology increases with the potential benefits derived from its use [62]. As users create individual content on rating portals, the question of *credibility* regarding this content in particular and the evaluation portal as a whole is essential to assess the *performance expectancy* [63]. Therefore, an increase in credibility can lead to web-based portals being perceived as more useful [64,65]. Thus, we expect the following: a higher *credibility* leads to a higher *performance expectancy* toward PRWs (hypothesis 4).

Nevertheless, alternative factors can also exert impact on *performance expectancy*. Fedorko et al [66] extended the UTAUT in the area of electronic banking and demonstrated that social influence had a positive effect on the expected performance. For this reason, the corresponding hypothesis is as follows: a more positive *social influence* leads to a higher *performance expectancy* regarding PRWs (hypothesis 5).

Studies in different fields have shown that *performance expectancy* has a strong effect on the *behavioral intention* to use new or adapted technologies [67]. In line with published results in the field of technology acceptance (eg, Anderson and Schwager [48], Carlsson et al [68], and Marchewka and Kostiwa [69]), we propose the following: a higher *performance expectancy* leads to a higher *behavioral intention* to use PRWs (hypothesis 6).

**Figure 2:**
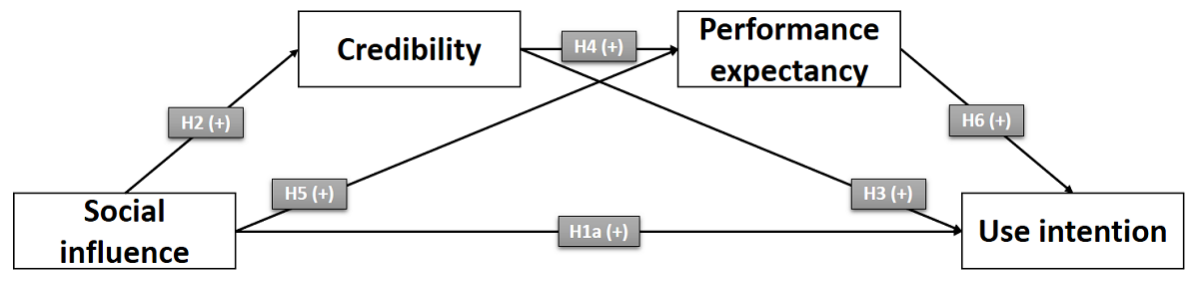
Conceptual serial mediation model. H: hypothesis.

Figure 2 shows the proposed relationships between the constructs and, thus, the proposed influencing chain.

#### Moderators of the Impact of Social Influence on the Behavioral Intention to Use PRWs

*Age* and *gender* are 2 demographic characteristics that have been discovered to affect the *behavioral intention* to use a new or adapted technology [70-73]. For this reason, we propose that the *age* of the participants moderates the effects between *social influence* and the *behavioral intention* to use PRWs as well as between *social influence* and *credibility* of PRWs (hypothesis 7a) and that the *gender* of the participants moderates the effects between *social influence* and the *behavioral intention* to use PRWs as well as between *social influence* and *credibility* of PRWs (hypothesis 7b).

In addition to these demographic characteristics, psychographic characteristics could also moderate the proposed effects. *eHealth literacy* describes the extent to which individuals are able to distinguish useful health-related information on the internet from less useful information [74]. Even though it was shown that *eHealth literacy* seems to exert little influence under certain conditions [75,76], it was also discovered that a high *eHealth literacy* can lead to an increase in the use behavior regarding health-related digital and mobile technologies [77,78]. In the context of PRWs, Schulz and Rothenfluh [79] observed that a higher *eHealth literacy* may mitigate the strength regarding the impact of individual reviews. This result shows that individuals with a high *eHealth literacy* seem to be less impressionable, especially in the medical web-based rating environment. On the basis of these results and assuming that individuals with a high *eHealth literacy* know where to find health-related information on the web, it is expected that a high *eHealth literacy* weakens the effects of our research model. For this reason, we propose that a high *eHealth literacy* weakens the effects between *social influence* and the *behavioral intention* to use PRWs as well as between *social influence* and *credibility* of PRWs (hypothesis 8a).

In addition to *eHealth literacy*, the level of skepticism regarding web-based reviews may also weaken the proposed effects in our research model. Consumer skepticism toward marketing and communication activities has a long research history [80]. Particularly in the digital environment, it has been shown that the level of skepticism toward web-based information can play a significant role in different types of decision-making [81,82]. A distinctive expression of consumer skepticism is *review skepticism*, which can be defined as mistrust toward electronic word of mouth in the context of web-based reviews [83]. In our study, review skepticism was conceptualized as a dispositional form of skepticism and not a situational one [81]. As it can be assumed that individuals who reveal a higher level of *review skepticism* are even more critical toward information on PRWs, we postulate that a high level of *review skepticism* weakens the effects between s*ocial influence* and the *behavioral intention* to use PRWs as well as between *social influence* and *credibility* of PRWs (hypothesis 8b).

To check the hypotheses, a mixed methods approach was applied by conducting 2 studies with different target samples and an experimental as well as cross-sectional study approach. Both of the studies are explained in detail in the following sections.

### Study 1

#### Methods

##### Study Design and Measures

To test the proposed hypothesized model as depicted in Figure 2, in a first step of our research endeavor, study 1 was conducted by performing a web-based questionnaire–based experiment with a between-subject design. Through randomized experimental manipulation, study participants were assigned to either the experimental group or the control group. After entering sociodemographic data, both groups received the following information: “Physician Rating Websites offer health care consumers the opportunity to evaluate their doctor anonymously. These evaluations could assist future or potential patients in decision-making regarding their future medical care.” In addition to that, the experimental group was asked to imagine that someone who influences their behavior or is important to them or whose opinion is appreciated has recommended the use of PRWs, whereas the control group did not receive this additional information. After that first part, respondents were asked to evaluate their perceived credibility and performance expectancy regarding PRW use as well as their behavioral intention to use PRWs.

The web-based questionnaire used in the experimental setting of study 1 was based on the adoption of established and validated scales [24,26,49,84-88]. The item wording of the questionnaire can be found in Multimedia Appendix 1 [24,26,49,84-88]. All items used were translated and back translated by both an English and a German native speaker who each had fluent language skills in their respective foreign language. To identify potential ambiguity in wording, a pretest with 20 participants was performed. After slight modifications based on the pretest results, the final version of the questionnaire was developed, which was then used for the main study.

##### Procedure

Data collection was performed using the web-based survey tool Google Forms. Respondents were invited to participate through various web-based channels such as email or social media (snowball sampling), and the survey was conducted over a period of 1 month, from April 15, 2019, to May 14, 2019.

##### Ethical Considerations

In Austria, there is no requirement to go through an institutional review board or an ethical committee when conducting research with human participants. The questionnaire and study methods adhere to Austrian and European Union privacy laws. The study, as well as the questionnaire, has received clearance from a number of academics and university professors. Participants were properly informed and instructed about their voluntary participation in a web-based survey and were also given the reassurance that their data would be managed with strict confidentiality using acceptable methods, processes, and protocols. Individuals who freely decided to participate in the survey were notified about it in written form before and after they completed the questionnaire. The data were handled in a strictly confidential and anonymous manner.

##### Data Check

At the beginning of the questionnaire, participants were informed that there were no right or wrong answers and that they would serve the objective of the survey best if they answered the questions honestly to minimize the potential risk of common method bias [89]. Participants were also informed that their information would be handled with complete secrecy using appropriate techniques, processes, and protocols [90].

##### Measurement Models

SPSS Statistics (version 27; IBM Corp) was used to test the hypotheses. The data were analyzed using linear regressions [91]. In addition to that, regression-based mediation analyses [92] were conducted using the PROCESS macro for SPSS [93]. Of the 92 models included in the PROCESS macro, which are also depicted in the appendix of the corresponding book by Hayes [94], we identified model number 6 (*Y*=*i_y_*+*c*′*X*+*b_1_M_1_*+*b_2_M_2_*+*e_y_*) as the appropriate model for the mediation analysis [92,94]. Furthermore, we included 5000 bootstraps and chose a 95% CI. *Social influence* was defined as independent variable (*X*), the *behavioral intention* to use PRWs was defined as dependent outcome variable (*Y*), and *credibility* as well as *performance expectancy* were both conceptualized as mediators (*M_1_* and *M_2_*).

#### Results

##### Analysis of Used Concepts

The Cronbach α ranged from .88 (*performance expectancy*) to .98 (*behavioral* *intention* to use PRWs) for all multi-item measures. The evaluation of construct means shows a rather high c*redibility* (4.04, SD 1.43), *performance expectancy* (4.39, SD 1.55), and *behavioral* *intention* to use PRWs (4.15, SD 1.96). Table 1 provides a summary of the model construct and measures, including means, SDs, and Cronbach α calculation results.

Table 2 shows how the individual constructs correlate with each other. It is evident that there is a high correlation between *performance expectancy* and *behavioral* *intention* to use PRWs. In contrast, there is a low correlation between *credibility* and *performance expectancy* as well as between *credibility* and *behavioral intention* to use PRWs [95].

**Table 1:** Model constructs and measures.

Variable and item		Value, mean (SD)	Cronbach α	
**Credibility**				.96
	PRWs^a^ seem to be credible	4.54 (1.60)		
	PRWs seem to be reliable	4.49 (1.56)		
	PRWs seem to be honest	4.34 (1.65)		
	PRWs seem to be sincere	4.06 (1.64)		
	PRWs seem to be trustworthy	4.01 (1.65)		
	PRWs seem to have expert knowledge	3.77 (1.73)		
	PRWs seem to be experienced	3.72 (1.60)		
	PRWs seem to contain knowledgeable content	3.93 (1.72)		
	PRWs seem to be qualified	3.86 (1.65)		
	PRWs seem to be knowledgeable	3.73 (1.70)		
**Performance expectancy**				.88
	I think that PRWs are a useful tool	4.73 (1.70)		
	By using PRWs, I feel like I have more control over my health	4.22 (1.70)		
	Using PRWs will enhance my effectiveness in managing my health care	4.23 (1.80)		
**Intention to use PRWs**				.98
	I intend to use PRWs in the future	4.14 (2.01)		
	I will try to use PRWs	4.16 (1.98)		
	I plan to use PRWs	4.13 (2.02)		

^a^PRW: physician rating website.

**Table 2:** Construct correlations^a^.

Variable	Value, mean (SD)	Correlations (2-sided; 95% CI)		
		1	2	3
Credibility	4.04 (1.43)	1	0.29 (0.16-0.42)	0.23 (0.10-0.36)
Performance expectancy	4.39 (1.55)	0.29 (0.16-0.42)	1	0.53 (0.42-0.62)
Intention to use PRWs^b^	4.15 (1.96)	0.23 (0.10-0.36)	0.53 (0.42-0.62)	1

^a^All correlations have a *P* value of <.001.

^b^PRW: physician rating website.

##### Participant Characteristics

A total of 194 participants took part in the study. As the questionnaire was primarily sent out in the university environment, it can be assumed that most study participants were members (students and employees) of a midsized Austrian university. Table 3 provides the sample description.

**Table 3:** Sample description (N=194).

Sociodemographic characteristics			Participants, n (%)
**Sex**			
	Female	106 (54.6)	
	Male	88 (45.4)	
**Age (years)**			
	20 to 24	31 (16)	
	25 to 29	18 (9.3)	
	30 to 34	26 (13.4)	
	35 to 39	56 (28.9)	
	40 to 44	15 (7.7)	
	45 to 49	2 (1)	
	50 to 54	8 (4.1)	
	55 to 59	13 (6.7)	
	≥60	20 (10.3)	
**Education**			
	Compulsory education	20 (10.3)	
	Vocational secondary education	9 (4.6)	
	Apprenticeship	29 (14.9)	
	High school	29 (14.9)	
	University degree	80 (41.2)	
	No answer	27 (13.9)	
**Marital status**			
	Single	43 (22.2)	
	Close-partnered	68 (35.1)	
	Married	42 (21.6)	
	Divorced	5 (2.6)	
	No answer	36 (18.6)	
**Occupation**			
	Salaried employee	102 (52.6)	
	Unemployed	3 (1.5)	
	Self-employed	8 (4.1)	
	In training (pupil or student)	47 (24.2)	
	Retired	3 (1.5)	
	No answer	31 (16)	
**Area of living**			
	Urban	116 (59.8)	
	Rural	74 (38.1)	
	No answer	4 (2.1)	

##### Test of Hypotheses

**Figure 3:**
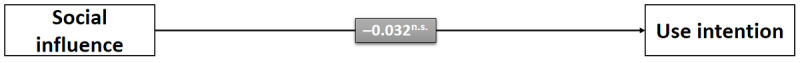
Direct effect of social influence on the behavioral intention to use physician rating websites (study 1). *P<.05; **P<.01; ***P<.001; n.s.: not significant.

In hypothesis 1, we propose that a positive *social influence* leads to a higher *behavioral intention* to use PRWs. This relationship could not be confirmed by the data as no direct effect of *social influence* on the *behavioral intention* to use PRWs could be found (*b*=−0.032; *P=*.91; SE −0.008; *t*_1_=−0.114). For this reason, hypothesis 1 has to be rejected. Figure 3 shows the direct effect of *social influence* on the *behavioral intention* to use PRWs.

In hypothesis 1a, we assume that, in the mediation model, the direct effect between *social influence* and the *behavioral intention* to use PRWs gets weaker compared with the single linear regression. However, as the direct effect between *social influence* and the *behavioral intention* to use PRWs could not be demonstrated in hypothesis 1, it cannot be assumed that there is a direct effect between the independent and the dependent variable in the mediation model. This assumption is confirmed by the data as there is no significant direct effect between *social influence* and the *behavioral intention* to use PRWs in the mediation model (*b*=−0.037; *P=*.88). For this reason, hypothesis 1a has to be rejected.

Hypotheses 2 to 6 describe the indirect effect between *social influence* and the *behavioral intention* to use PRWs through 2 mediators (ie, *credibility* and *performance expectancy*). In this context, hypothesis 2 suggests that a positive *social influence* leads to a higher *credibility* of PRWs. The results referring to this assumption confirm that a positive *social influence* led to an increased *credibility* of PRWs (*b*=0.572; *P=*.005). This shows that, for the experimental group, PRWs seemed to be more credible than for the control group. Thus, hypothesis 2 is confirmed.

Referring to hypothesis 3, it is believed that a higher *credibility* also leads to a higher *behavioral intention* to use PRWs. However, this relationship could not be observed (*b*=−0.123; *P=*.18). For this reason, hypothesis 3 has to be rejected.

Hypothesis 4 examines the influence of *credibility* on the *behavioral intention* to use PRWs. The analysis in the mediation model confirms the expected relationship. Higher *credibility* led to an increase in *performance expectancy* (*b*=0.340; *P*<.001). For this reason, hypothesis 4 is confirmed.

Hypothesis 5 assumes that a stronger *social influence* also leads to a direct increase in *performance expectancy*. This relationship could not be observed (*b*=−0.298; *P*=.17). For this reason, hypothesis 5 has to be rejected.

**Figure 4:**
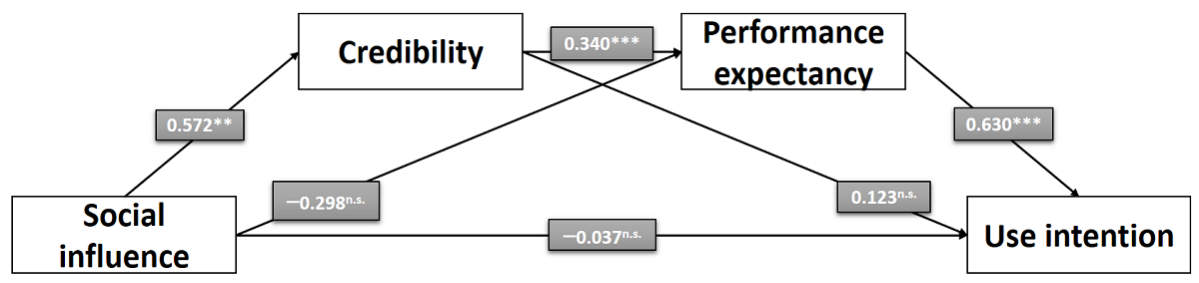
Serial mediation model (study 1). *P<.05; **P<.01; ***P<.001; n.s.: not significant.

Hypothesis 6 examines the influence of *performance expectancy* on the *behavioral intention* to use PRWs. The analysis in the mediation model confirms the expected relationship. Higher *performance expectancy* led to a substantial increase in the *behavioral intention* to use PRWs (*b*=0.630; *P*<.001). Thus, hypothesis 6 is confirmed. Figure 4 shows the mediation model including estimates and *P* values for the linear regressions. In addition to that, Table 4 shows the detailed outcomes for our proposed mediation model, including model summary, SEs, and 2-tailed *t* test and *P* values.

Nevertheless, to check the external validity of the proposed effect chain and replicate the findings, another study was conducted. To assure external validity, another research setting (ie, a cross-sectional study instead of an experimental setting) was applied. In addition, the target sample of the second study should reflect a broader range of individuals, representing the general (web-based) population in a better way, and we intended to use a larger sample size instead of the small sample of 194 largely university members (students and employees) from a southern Austrian university.

**Table 4:** Serial mediation model outcomes.

Outcome variable and variable		Coefficient b^a^ (SE; 95% CI)	*t* test (*df*)	*P* value
**Credibility^b^**				
	Constant	3.740 (0.147; 3.450 to 4.029)	25.457 (1)	<.001
	Social influence	0.572 (0.202; 0.175 to 0.970)	2.838 (1)	.005
**Performance expectancy^c^**				
	Constant	3.176 (0.326; 2.533 to 3.819)	9.743 (2)	<.001
	Social influence	−0.298 (0.218; −0.729 to 0.132)	−1.366 (2)	.17
	Credibility	0.340 (0.077; 0.189 to 0.491)	4.438 (2)	<.001
**Intention to use PRWs^d,e^**				
	Constant	0.904 (0.450; 0.018 to 1.790)	2.012 (3)	.046
	Social influence	−0.037 (0.247; −0.525 to 0.451)	−0.149 (3)	.88
	Credibility	0.123 (0.091; −0.056 to 0.301)	1.354 (3)	.18
	Performance expectancy	0.630 (0.082; 0.469 to 0.791)	7.730 (3)	<.001

^a^Regression coefficient.

^b^*R*=0.201; *R*^2^=0.040; *P*=.005.

^c^*R*=0.307; *R*^2^=0.095; *P*<.001.

^d^*R*=0.532; *R*^2^=0.283; *P*<.001.

^e^PRW: physician rating website.

### Study 2

#### Methods

##### Study Design and Measures

In study 2, the constructs were once again measured using existing and validated measures [24,26,49,84-88,96-98]. The questionnaire used in study 2 can be found in Multimedia Appendix 2 [24,26,49,84-88,96-98]. Again, all items were translated and back translated by native English and German speakers who each had fluent language skills in their respective foreign language. The scale options in all variables of interest, apart from the demographic ones, ranged from 1 (“strongly disagree”) to 7 (“strongly agree”).

In contrast to the experimental research applied in study 1, the cross-sectional research approach applied in study 2 is less appropriate to indicate the direction of proposed effects. Thus, the direction of effects is not specified, and study models with alternative effect chains could be set up. However, the use of cross-sectional data is quite common in the analysis of mediation effects as cross-sectional studies are often the only feasible approach for certain topics [99-109] and for targeting larger sample sizes as we intended in study 2. Thus, the methodological approach of our cross-sectional study, which was used in study 2, is in line with existing literature using cross-sectional data to test hypothesized models [99-109]. Both of our studies apply different methodological approaches but lead to comparable results. This agreement in the results supports the validity of the proposed mediation model. However, it has been mentioned previously that cross-sectional data alone may not be suitable to confirm or reject hypotheses proposing a causal direction of effects. To act cautiously and anticipate points of criticism in this vein, we decided not to report in the Results section of study 2 whether hypotheses were confirmed or had to be rejected but to report whether the data were in line with the proposed hypotheses or not.

##### Procedure

Data collection was carried out in February 2021 and March 2021. The data were gathered using a web-based panel (Clickworker GmbH), which is a research crowdsourcing platform comparable with Amazon’s Mechanical Turk.

##### Ethical Considerations

The study methodology, questionnaire, and survey instrument adhered to European Union and Austrian privacy laws. The questionnaire did not address any sensitive subjects, and the evaluation process precluded drawing any conclusions about the survey respondents. The questions were kept generic, and there was never any risk of harm from answering them. None of the panel members were asked for any sensitive information, and they all agreed to the data gathering. As mentioned previously, the study was conducted using the crowdsourcing platform Clickworker. This platform has pledged to abide by the General Data Protection Regulation standards and has obtained ISO 27001 certification. Before being approved, all projects and orders must pass an auditing procedure. In this process, professional staff members review for any survey issues, requests for personal information, and instances of discriminatory or unethical content. Orders requesting personal information or containing offensive or unethical content are not accepted. Participants were given the proper information and instructions on their voluntary participation in a web-based survey as well as the knowledge that their data would be handled with utmost secrecy. The processing of the data was completely private and anonymous.

##### Data Check

The constructs included in study 2 were self-reported measures that are associated with the risk of common method bias [110]. To minimize the potential risk of common method bias, we informed participants that there were no right or wrong answers and that they would serve the purpose of the survey best if they answered the questions as honestly as possible [89]. In addition to that, participants were informed that their data would be treated with absolute confidentiality using suitable methods, procedures, and protocols [90].

Furthermore, we ran multiple pretests and eliminated unclear or imprecise data sets [111]. Using the survey tool LimeSurvey (LimeSurvey GmbH), the data were thoroughly checked for inconsistent answer patterns, flatliners, and very short answer times. In this context, we eliminated questionnaires from respondents who used descending or ascending numerical sequences for the items throughout the questionnaire (inconsistent answer patterns), consistently responded with the same answer (flatliners), or completed the questionnaire in <265 seconds (very short answer times). The minimum response time for answering the questionnaire was pretested by the authors.

By incorporating 3 security levels in the web-based questionnaire, we followed guidelines to reduce validity concerns when using crowdsourcing platforms (eg, see Aguinis et al [112]). First, logic tasks and attention tests were used to verify that survey participants’ attention and integrity were maintained. Participants had to solve a mathematical equation to verify that they were human and that they were eligible before they could begin answering the questions. In addition to that, there was a check for attention. See Multimedia Appendix 3 for an example equation and the attention check. To verify that participants had read the introductory text of the third question group, they had to pick a specific answer choice in a specific question as part of this attention check (see Oppenheimer et al [113] and Kung et al [114]). Finally, each participant was assigned a cookie to prevent them from taking part again.

There were no missing data in the survey as the questionnaire instrument was not set up to allow for unanswered questions.

##### Measurement Models

SPSS Statistics (version 27; IBM Corp) was used to test the hypotheses. The data were analyzed using linear regressions [91]. In addition to that, regression-based mediation analyses [92] were conducted using the PROCESS macro for SPSS [93]. Of the 92 models included in the PROCESS macro, which are also depicted in the appendix of the corresponding book by Hayes [93], we again identified model number 6 (*Y*=*i_y_*+*c′X*+*b_1_M_1_*+*b_2_M_2_*+*e_y_*) for the mediation analysis and model number 8 (*Y*=*i_y_*+*c′_1_X*+*c′_2_W*+*c′_3_XW*+*b_1_M_1_*+*b_2_M_2_*+*e_y_*) for the moderated mediation analysis [92,94] as the appropriate models in our case. Furthermore, we included 5000 bootstraps and chose a 95% CI. *Social influence* was defined as independent variable (*X*), the *behavioral intention* to use PRWs was defined as dependent outcome variable (*Y*), and *credibility* as well as *performance expectancy* both were conceptualized as mediators (*M_1_* and *M_2_*). *Age*, *gender*, and *eHealth literacy* as well as *review skepticism* acted as moderator variables in our models. The analyses were performed in a hierarchical order starting with the basic mediation model. After that, the proposed moderators were included one after the other.

#### Results

##### Analysis of Used Concepts

The Cronbach α ranged from.79 (*performance expectancy*) to.97 (*behavioral* *intention* to use PRWs) for all multi-item measures. The evaluation of construct means shows a rather high *credibility* (4.44, SD 1.11) and *behavioral* *intention* to use PRWs (4.14, SD 1.90). The mean value of *performance expectancy* (3.62, SD 1.26) is slightly above the midpoint of the scale. However, on average, *social influence* (2.47, SD 1.56) in the domain of PRWs in real life (as opposed to the experimental manipulation of social influence in study 1) seems to be rather low. Table 5 provides a summary of the model construct and measures, including means, SDs, and Cronbach α calculation results.

Table 6 shows how the individual constructs correlate with each other. It is evident that there is a high correlation between *performance expectancy* and *behavioral* *intention* to use PRWs. By contrast, there is a low correlation between *social influence* and *credibility*. The remaining constructs are characterized by a medium correlation with each other [95].

**Table 5:** Model constructs and measures.

Variable and item			Value, mean (SD)		Cronbach α
**Social influence**			2.47 (1.56)		.96
	People who influence my behavior think that I should use PRWs^a^	2.39 (1.57)			
	People who are important to me think that I should use PRWs	2.49 (1.61)			
	People whose opinion I value think that I should use PRWs	2.53 (1.66)			
**Credibility**			4.44 (1.11)		.92
	PRWs seem to be credible	4.50 (1.76)			
	PRWs seem to be reliable	4.37 (1.22)			
	PRWs seem to be trustworthy	4.44 (1.21)			
**Performance expectancy**			3.62 (1.26)		.79
	I think that PRWs are a useful tool	4.99 (1.50)			
	By using PRWs, I feel like I have more control over my health	3.14 (1.71)			
	Using PRWs will enhance my effectiveness in managing my health care	3.64 (1.67)			
	Overall, PRWs will be useful in managing my health care	2.70 (1.55)			
**Intention to use PRWs**			4.14 (1.90)		.97
	I intend to use PRWs in the future	4.24 (1.97)			
	I will try to use PRWs	4.18 (1.92)			
	I plan to use PRWs	4.00 (2.00)			

^a^PRW: physician rating website.

**Table 6:** Construct correlations^a^.

Variable	Value, mean (SD)	Correlations (2-sided; 95% CI)			
		1	2	3	4
Social influence	2.47 (1.56)	1	0.29 (0.21-0.37)	0.49 (0.43-0.56)	0.41 (0.34-0.48)
Credibility	4.44 (1.11)	0.29 (0.21-0.37)	1	0.50 (0.42-0.56)	0.47 (0.40-0.54)
Performance expectancy	3.62 (1.26)	0.50 (0.43-0.56)	0.50 (0.43-0.56)	1	0.59 (0.53-0.64)
Intention to use PRWs^b^	4.14 (1.90)	0.41 (0.34-0.48)	0.47 (0.40-0.54)	0.59 (0.53-0.64)	1

^a^All correlations have a *P* value of <.001.

^b^PRW: physician rating websites.

##### Participant Characteristics

A total of 852 participants from Austria took part in the study, with 334 (39.2%) of them being eliminated as they were not able to pass the manipulation check (239/334, 71.6%) or were characterized by an implausible response behavior or insufficient answer time (95/334, 28.4%). See Multimedia Appendix 4 for a graphical data cleansing description. This data cleansing mechanism resulted in a total of 518 survey participants who form the calculation sample for this study. Table 7 provides the sample description.

**Table 7:** Sample description (N=518).

Sociodemographic characteristics			Participants, n (%)
**Sex**			
	Female	289 (55.8)	
	Male	227 (43.8)	
	Intersex	2 (0.4)	
**Age (years)**			
	15 to 19	62 (12)	
	20 to 24	141 (27.2)	
	25 to 29	101 (19.5)	
	30 to 34	75 (14.5)	
	35 to 39	64 (12.4)	
	40 to 44	32 (6.2)	
	45 to 49	14 (2.7)	
	50 to 54	13 (2.5)	
	55 to 59	8 (1.5)	
	≥60	8 (1.5)	
**Education**			
	Compulsory education	27 (5.2)	
	Vocational secondary education	55 (10.6)	
	Apprenticeship	72 (13.9)	
	High school	214 (41.3)	
	University degree	150 (29)	
**Marital status**			
	Single	203 (39.2)	
	Close-partnered	218 (42.1)	
	Married	87 (16.8)	
	Divorced	10 (1.9)	
**Occupation**			
	Salaried employee	245 (47.3)	
	Unemployed	43 (8.3)	
	Self-employed	47 (9.1)	
	In training (pupil or student)	175 (33.8)	
	Retired	8 (1.5)	
**Area of living**			
	Urban	322 (62.2)	
	Rural	196 (37.8)	

##### Test of Hypotheses

**Figure 5:**
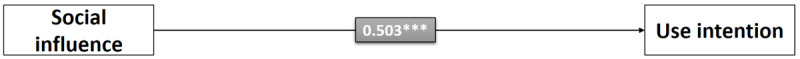
Direct effect of social influence on the behavioral intention to use physician rating websites (study 2). *P<.05; **P<.01; ***P<.001; n.s.: not significant.

In hypothesis 1, we propose that a positive social influence leads to a higher behavioral intention to use PRWs. This relationship is in line with the data as it was shown that respondents whose social environment influenced them to a greater extent to use those websites seemed to have a higher behavioral intention to use PRWs than respondents who were less influenced to use PRWs by their social environment (*b*=0.503; *P*<.001; SE 0.049; *t*_1_=10.197). Thus, the data were in line with hypothesis 1. Figure 5 shows the direct effect of social influence on the behavioral intention to use PRWs. However, in hypothesis 1a, we assume that the impact of *social influence* on *behavioral intention* to use PRWs is partly explained by the mediator variables *credibility* and *performance expectancy*. Therefore, this conclusion would lead to the direct impact of *social influence* on the *behavioral intention* to use PRWs being weaker in the mediation model than in the simple linear regression. In this context, the data are in line with hypothesis 1a as the direct effect of *social influence* on the *behavioral intention* to use PRWs in the mediation model was weaker (*b*=0.177; *P*<.001) than the effect measured in the simple linear regression (*b*=0.410; *P*<.001). On the basis of these results, the data were in line with hypothesis 1a.

Hypotheses 2 to 6 describe the indirect effect between *social influence* and the *behavioral intention* to use PRWs through 2 mediators (ie, *credibility* and *performance expectancy*). In this context, hypothesis 2 suggests that a positive *social influence* leads to a higher *credibility* of PRWs. The results referring to this assumption suggest that, for respondents whose social environment influenced them to a greater extent to use those websites, PRWs seemed to be more credible than for respondents who were less influenced to use PRWs by their social environment (*b*=0.208; *P*<.001). Thus, the data are in line with hypothesis 2.

Hypothesis 3 assumes that higher *credibility* also increases the *behavioral intention* to use PRWs. Study participants who indicated that PRWs were more credible also seemed to have a higher *behavioral intention* to use PRWs (*b*=0.402; *P*<.001). For this reason, hypothesis 3 is in line with the data.

Referring to hypothesis 4, it is believed that a higher *credibility* also leads to a higher *performance expectancy* toward PRWs. Results show that respondents reporting a higher *credibility* regarding PRWs also reported a higher *performance expectancy* toward PRWs (*b*=0.431; *P*<.001). On the basis of these results, hypothesis 4 is in line with the data.

Hypothesis 5 assumes that a stronger *social influence* also leads to a direct increase in *performance expectancy*. In this context, it could be observed that respondents whose social environment influenced them to a greater extent to use those websites also reported a higher *performance expectancy* regarding PRWs (*b*=0.312; *P*<.001). For this reason, hypothesis 5 is in line with the data.

**Figure 6:**
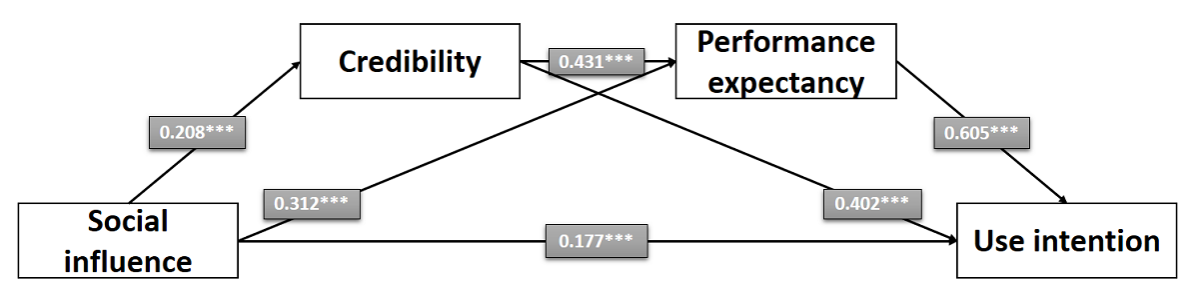
Serial mediation model (study 2). *P<.05; **P<.01; ***P<.001; n.s.: not significant.

Hypothesis 6 examines the influence of *performance expectancy* on the *behavioral intention* to use PRWs. The analysis in the mediation model showed that respondents who reported a higher *performance expectancy* regarding PRWs also reported a higher *behavioral intention* to use PRWs (*b*=0.605; *P*<.001). Thus, hypothesis 6 is in line with the data. Figure 6 shows the mediation model, including estimates and *P* values for the linear regressions. In addition to that, Table 8 shows the detailed outcomes for our proposed mediation model, including model summary, SEs, and *t* test and *P* values.

Hypotheses 7 and 8 control for potential moderator effects in our model. In hypothesis 7a, we suggest that the *age* of the participants affects the effects between *social influence* and the *behavioral intention* to use PRWs as well as between *social influence* and *credibility* of PRWs. To test this hypothesis, we created a subsample consisting of 2 age groups. Respondents aged <39 years were assigned to the younger age group (443/518, 85.5%), and respondents aged ≥39 years were assigned to the older age group (75/518, 14.5%). No significant interaction effects could be demonstrated when examining *age* as a potential moderator variable. For this reason, hypothesis 7a had to be rejected. However, it could be shown that higher *age* has a negative influence on *performance expectancy* (*b*=−0.501; *P*=.04). Table 9 summarizes the results of the corresponding moderated mediation model.

In hypothesis 7b, we suggest that the *gender* of the participants affects the effects between *social influence* and the *behavioral intention* to use PRWs as well as between *social influence* and *credibility* of PRWs. To examine this assumption, we excluded participants who reported that they belonged to the *intersex* category. The exclusion was made as the response rate of this population group was very low (2/518, 0.4%). No significant interaction effects could be demonstrated when examining *gender* as a potential moderator variable. For this reason, hypothesis 7b had to be rejected. Table 10 summarizes the results of the corresponding moderated mediation model.

In hypothesis 8a, we suggest that a high level of *eHealth literacy* weakens the effects between *social influence* and the *behavioral intention* to use PRWs as well as between *social influence* and *credibility* of PRWs. Accordingly, a high *eHealth literacy* should weaken the direct as well as indirect effect in the moderated mediation model. Even though in the model it was shown that *eHealth literacy* had a positive effect on *credibility* (*b*=0.266; *P*=.002), this effect could not be observed between *eHealth literacy* and the *behavioral intention* to use PRWs (*b*=0.173; *P*=.17). In addition to that, the proposed interaction effects could also not be observed as *eHealth literacy* did not exert significant influence on the effect between *social influence* and *credibility* (*b*=0.018; *P*=.55) or the effect between *social influence* and the *behavioral intention* to use PRWs (*b*=0.005; *P*=.92). For this reason, hypothesis 8a had to be rejected. Table 11 summarizes the results of the corresponding moderated mediation model.

Finally, in hypothesis 8b, we suggest that a high level of *review skepticism* weakens the effects between *social influence* and the *behavioral intention* to use PRWs as well as between *social influence* and *credibility* of PRWs. Accordingly, a high level of *review skepticism* should weaken the direct as well as indirect effect in the moderated mediation model. Even though in the model it was shown that r*eview skepticism* had a negative effect on *credibility* (*b*=**−**0.254; *P*<.001), this effect could not be observed between *review skepticism* and the *behavioral intention* to use PRWs (*b*=**−**0.111; *P*=.24). In addition to that, the proposed interaction effects could also not be observed as *review skepticism* did not exert significant influence on the effect between *social influence* and *credibility* (*b*=0.025; *P*=.25) or the effect between *social influence* and the *behavioral intention* to use PRWs (*b*=0.017; *P*=.58). For this reason, hypothesis 8b had to be rejected. Table 12 summarizes the results of the corresponding moderated mediation model.

**Table 8:** Serial mediation model outcomes.

Outcome variable			Coefficient b^a^ (SE; 95% CI)		*t* test (*df*)		*P* value
**Credibility^b^**							
	Constant	3.925 (0.088; 3.751 to 4.098)		44.393 (1)		<.001	
	Social influence	0.208 (0.030; 0.148 to 0.267)		6.845 (1)		<.001	
**Performance expectancy^c^**							
	Constant	0.935 (0.181; 0.580 to 1.290)		5.172 (2)		<.001	
	Social influence	0.312 (0.030; 0.254 to 0.370)		10.590 (2)		<.001	
	Credibility	0.431 (0.041; 0.351 to 0.512)		10.516 (2)		<.001	
**Intention to use PRWs^d,e^**							
	Constant	−0.275 (0.274; −0.814 to 0.264)		−1.003 (3)		.32	
	Social influence	0.177 (0.048; 0.082 to 0.271)		3.665 (3)		<.001	
	Credibility	0.402 (0.067; 0.271 to 0.533)		6.012 (3)		<.001	
	Performance expectancy	0.605 (0.065; 0.477 to 0.734)		9.283 (3)		<.001	

^a^Regression coefficient.

^b^*R*=0.289; *R*^2^=0.083; *P*<.001.

^c^*R*=0.615; *R*^2^=0.378; *P*<.001.

^d^*R*=0.637; *R*^2^=0.406; *P*<.001.

^e^PRW: physician rating website.

**Table 9:** Results of the moderated mediation analyses with age as moderator.

Outcome variable			Coefficient b^a^ (SE; 95% CI)	*t* test (*df*)		*P* value
**Credibility^b^**						
	Constant	3.992 (0.097; 3.802 to 4.182)		41.299 (3)	<.001	
	Social influence	0.194 (0.033; 0.130 to 0.258)		5.952 (3)	<.001	
	Age	−0.383 (0.239; −0.853 to 0.087)		−1.600 (3)	.11	
	Interaction: (social influence × age)	0.071 (0.091; −0.108 to 0.249)		0.777 (3)	.44	
**Performance expectancy^c^**						
	Constant	2.714 (0.099; 2.520 to 2.908)		27.438 (3)	<.001	
	Social influence	0.383 (0.033; 0.318 to 0.449)		11.504 (3)	<.001	
	Age	−0.501 (0.245; −0.982 to −0.020)		–2.045 (3)	.04	
	Interaction: (social influence × age)	0.098 (0.093; −0.085 to 0.281)		1.054 (3)	.29	
**Intention to use PRWs^d,e^**						
	Constant	−0.124 (0.285; −0.682 to 0.435)		−0.435 (5)	.66	
	Social influence	0.152 (0.050; 0.053 to 0.251)		3.021 (5)	.003	
	Credibility	0.397 (0.067; 0.266 to 0.528)		5.942 (5)	<.001	
	Performance expectancy	0.596 (0.065; 0.468 to 0.725)		9.135 (5)	<.001	
	Age	−0.650 (0.331; −1.300 to 0.001)		−1.961 (5)	.05	
	Interaction: (social influence × age)	0.189 (0.125; −0.058 to 0.435)		1.503 (5)	.13	

^a^Regression coefficient.

^b^*R*=0.299; *R*^2^=0.090; *P*<.001.

^c^*R*=0.503; *R*^2^=0.253; *P*<.001.

^d^*R*=0.640; *R*^2^=0.410; *P*<.001.

^e^PRW: physician rating website.

**Table 10:** Results of the moderated mediation analyses with gender as moderator.

Outcome variable			Coefficient b^a^ (SE; 95 CI)		*t* test (*df*)		*P* value
**Credibility^b^**							
	Constant	3.916 (0.271; 3.384 to 4.448)		14.458 (3)		<.001	
	Social influence	0.222 (0.096; 0.034 to 0.410)		2.317 (3)		.02	
	Gender	0.002 (0.181; −0.353 to 0.357)		0.011 (3)		.99	
	Interaction: (social influence × gender)	−0.009 (0.061; −0.129 to 0.112)		−0.145 (3)		.88	
**Performance expectancy^c^**							
	Constant	2.539 (0.278; 1.992 to 3.085)		9.125 (3)		<.001	
	Social influence	0.454 (0.098; 0.260 to 0.647)		4.608 (3)		<.001	
	Gender	0.058 (0.186; −0.306 to 0.422)		0.313 (3)		.75	
	Interaction: (social influence × gender)	−0.034 (0.063; −0.158 to 0.090)		−0.542 (3)		.59	
**Intention to use PRWs^d,e^**							
	Constant	0.250 (0.445; −0.623 to 1.124)		0.563 (5)		.57	
	Social influence	0.169 (0.134; −0.096 to 0.420)		−1.233 (5)		.22	
	Credibility	0.407 (0.065; 0.280 to 0.535)		6.255 (5)		<.001	
	Performance expectancy	0.599 (0.062; 0.476 to 0.721)		9.609 (5)		<.001	
	Gender	−0.364 (0.240; −0.836 to 0.108)		−1.514 (5)		.13	
	Interaction: (social influence × gender)	0.078 (0.083; −0.144 to 0.180)		0.216 (5)		.83	

^a^Regression coefficient.

^b^*R*=0.290; *R*^2^=0.084; *P*<.001.

^c^*R*=0.495; *R*^2^=0.245; *P*<.001.

^d^*R*=0.643; *R*^2^=0.413; *P*<.001.

^e^PRW: physician rating website.

**Table 11:** Results of the moderated mediation analyses with eHealth literacy as moderator.

Outcome variable		Coefficient b^a^ (SE; 95% CI)	*t* test (*df*)	*P* value
**Credibility^b^**				
	Constant	2.463 (0.490; 1.501 to 3.425)	5.031 (3)	<.001
	Social influence	0.111 (0.170; −0.224 to 0.445)	0.649 (3)	.52
	eHealth literacy	0.266 (0.087; 0.095 to 0.438)	3.056 (3)	.002
	Interaction: (social influence × eHealth literacy)	0.018 (0.030; −0.042 to 0.078)	0.592 (3)	.55
**Performance expectancy^c^**				
	Constant	1.920 (0.519; 0.901 to 2.940)	3.700 (3)	<.001
	Social influence	0.342 (0.181; −0.012 to 0.698)	1.899 (3)	.06
	eHealth literacy	0.129 (0.092; −0.053 to 0.310)	1.394 (3)	.17
	Interaction: (social influence × eHealth literacy)	0.011 (0.032; −0.052 to 0.074)	0.338 (3)	.74
**Intention to use PRWs^d,e^**				
	Constant	−1.030 (0.719; −2.442 to 0.382)	−1.433 (5)	.15
	Social influence	0.165 (0.244; −0.315 to 0.644)	0.674 (5)	.50
	Credibility	0.355 (0.069; 0.220 to 0.490)	5.158 (5)	<.001
	Performance expectancy	0.601 (0.065; 0.474 to 0.729)	9.267 (5)	<.001
	eHealth literacy	0.173 (0.126; −0.074 to 0.420)	1.378 (5)	.17
	Interaction: (social influence × eHealth literacy)	0.005 (0.043; −0.081 to 0.090)	0.103 (5)	.92

^a^Regression coefficient.

^b^*R*=0.399; *R*^2^=0.159; *P*<.001.

^c^*R*=0.509; *R*^2^=0.260; *P*<.001.

^d^*R*=0.643; *R*^2^=0.414; *P*<.001.

^e^PRW: physician rating website.

**Table 12:** Results of the moderated mediation analyses with review skepticism as moderator.

Outcome variable			Coefficient b^a^ (SE; 95% CI)		*t* test (*df*)		*P* value
**Credibility^b^**							
	Constant	5.012 (0.288; 4.447 to 5.579)		17.403 (3)		<.001	
	Social influence	0.086 (0.092; −0.094 to 0.266)		0.936 (3)		.35	
	Review skepticism	−0.254 (0.066; −0.384 to 0.124)		−3.836 (3)		<.001	
	Interaction: (social influence × review skepticism)	0.025 (0.022; −0.018 to 0.068)		1.143 (3)		.25	
**Performance expectancy^c^**							
	Constant	3.158 (0.303; 2.564 to 3.752)		10.440 (3)		<.001	
	Social influence	0.307 (0.096; 0.118 to 0.496)		3.188 (3)		.002	
	Review skepticism	−0.126 (0.069; −0.262 to 0.011)		−1.809 (3)		.07	
	Interaction: (social influence × review skepticism)	0.022 (0.023; −0.023 to 0.067)		0.954 (3)		.34	
**Intention to use PRWs^d,e^**							
	Constant	0.277 (0.518; −0.740 to 1.294)		0.535 (5)		.59	
	Social influence	0.104 (0.131; −0.153 to 0.362)		0.797 (5)		.43	
	Credibility	0.382 (0.069; 0.247 to 0.516)		5.570 (5)		<.001	
	Performance expectancy	0.606 (0.065; 0.478 to 0.734)		9.287 (5)		<.001	
	Review skepticism	−0.111 (0.095; −0.298 to 0.075)		−1.174 (5)		.24	
	Interaction: (social influence × review skepticism)	0.017 (0.031; −0.044 to 0.078)		0.555 (5)		.58	

^a^Regression coefficient.

^b^*R*=0.365; *R*^2^=0.133; *P*<.001.

^c^*R*=0.501; *R*^2^=0.251; *P*<.001.

^d^*R*=0.639; *R*^2^=0.408; *P*<.001.

^e^PRW: physician rating website.

## Discussion

### Principal Findings

On the basis of the results of studies 1 and 2, *social influence* exerts a statistically significant impact on the *behavioral intention* to use PRWs [115,116]. However, in study 1, it was shown that this impact might only be exerted indirectly through the 2 mediator variables *credibility* and *performance expectancy*. Notwithstanding, when we tested the proposed chain of effects in study 2, we were able to reveal 2 further findings. On the one hand, we found a direct effect of *social influence* on the *behavioral intention* to use PRWs. However, this direct effect between *social influence* and the *behavioral intention* to use PRWs was significantly weakened in the mediation model. This result suggests that the direct effect between the independent and the dependent variable is at least partially explained by the 2 mediator variables [92,117]. Furthermore, the proposed indirect effect itself could again be observed in a significant expression.

In contrast to the successfully predicted direct and indirect effects between *social influence* and the *behavioral intention* to use PRWs, the proposed moderation effects could not be observed in our moderated mediation model. Even though *eHealth literacy* and *review skepticism* seem to affect the *credibility* of PRWs, we were not able to observe a significant effect of *age*, *gender*, *eHealth literacy*, or *review skepticism* on the proposed mediation model.

These findings strongly support theories from the social psychological perspective, theories of technology acceptance, and theories from the economic perspective. From a social psychological perspective, the results of the 2 studies support both the TRA and its extension, the TPB. Both theories serve as a basis for the factor model studied. However, the TRA can also generally be reconstructed by means of the factors investigated. As described in the Introduction, *social influence* is a continuation of *subjective norms* [25]. *Credibility* as well as *performance expectancy* may be considered as determinants of *attitude* toward behavior, and *behavioral intention* is a construct that is incorporated as a result of *attitudes* and *subjective norms* within the TRA [9]. The 2 studies also show a generally similar influencing chain. Thus, the results are in line with a large number of empirical studies in the field of social psychological theories (eg, Gotch and Hall [118], Fishbein [119], Ajzen et al [120], Lada et al [121], and Buttle and Bok [122]) and provide evidence for the validity of the TRA.

The results of the 2 studies also support theories of technology acceptance as it was shown that *social influence* affects the *behavioral intention* to use PRWs indirectly (studies 1 and 2) and directly (study 2). Furthermore, the impact of *performance expectancy* on the dependent variable could also be demonstrated in the factor model. Even though not all technology acceptance variables were considered in the factor model, these studies still provide evidence for the validity of the respective influencing paths included in the UTAUT.

Finally, the findings of both studies strongly support the TIE as they show that individuals in our study samples built their decision for or against the *behavioral intention* to use PRWs on *social influence* through *credibility* and *performance expectancy*. This outcome indicates that individuals may base their decision for or against the use of these web-based platforms at least partly on factors other than the performance quality of PRWs. In addition to that, our results support the similarity effect as we were able to show that advice from a person with a high similarity could lead to an increased *behavioral intention* to use PRWs.

Furthermore, a path model in the form examined in this study has never been tested in the context of PRWs. Therefore, the added value of the studies also lies in developing practical implications from the relationships that could specifically increase the degree of use of PRWs (eg, through the targeted use of social influence by social media influencers [SMIs]).

### Limitations

Despite the significance of the findings, a number of limitations need to be considered. Particularly in web-based surveys, it is to be expected that research participants might be disinterested or respond in a one-sided manner. However, we tried our best to address and solve the issue of a possible common method bias. We attempted to minimize the risk of a potential common method bias by conducting information processes before and during the completion of the questionnaire by study participants. Furthermore, participants in a web-based survey may have a more thorough grasp of web-based issues. This might have resulted in a more prominent representation of the *behavioral intention* to use PRWs in the study population. In addition to that, by focusing on social influence as the only independent variable, we disregarded a number of alternative influencing variables. This may have led to a disproportionate impact of social influence on our study model. Finally, as was already explained in detail in the Methods section of study 2, it should be noted that a cross-sectional study, in contrast to the experimental study design of study 1, does not specify a direction of effects. In general, this means that the effect of the factors on each other could also be different from that proposed in the study model. An explanation could be that individuals tend to associate with people who share their attitudes and viewpoints (eg, see Bos et al [123]). Another explanation could be that people tend to exaggerate the degree to which their opinions and those of others are similar (eg, see Dunning et al [124]). We have made several attempts to address this criticism. First, the factor model of this study was built on established theories of social psychology and technology acceptance. In addition, we used a 2-step procedure to check the appropriateness of the proposed influencing chain. In a first step, we conducted a study applying an experimental setting to test the meaningfulness of the causal model. The cross-sectional study was the second step of our research endeavor. Although this approach lends considerable credence to the study model, there is still a certain residual risk that the influence paths of the integrated factors are not as interrelated as suggested.

### Conclusions and Practical Implications

The aim of the bipartite research endeavor was to investigate if and how *social influence* affects the *behavioral intention* to use PRWs. In study 1, the proposed indirect effect between *social influence* and the *behavioral intention* to use PRWs could be demonstrated. Moreover, in study 2, almost all of our hypotheses were in line with the data. The proposed serial mediation model provides evidence for the validity of both the TRA and the UTAUT. Moreover, we were able to observe the proposed similarity effect as a positive *social influence* led to a higher *credibility* of PRWs in both studies. In this context, the TIE can serve as a profound theoretical framework in explaining the relationships between the constructs. By categorizing health care services as credence goods, this theoretical approach can make valuable contributions in explaining the impact of *social influence* on the *credibility* of PRWs as well as on the *performance expectancy* and *behavioral intention* to use PRWs. The most obvious finding to emerge from both studies is that *social influence* seems to exert an impact on the *behavioral intention* to use PRWs. However, in particular, we showed that, under certain conditions, this impact seems not to be exerted directly but indirectly through *credibility* and *performance expectancy*. To sum up, the evidence from both studies suggests that *social influence* could increase the use rates of PRWs enormously. Bearing in mind that SMIs develop a kind of parasocial relationship with their followers [125,126], it might be conceivable that *social influence* is not just limited to friends and family but could also be exerted by SMIs in the domain of PRW use. From a marketing perspective, PRW providers could think of collaborating with SMIs to boost use of PRWs in the future. With the onset of the ongoing pandemic, and especially in times of lockdowns and reduced personal contacts, SMIs have increasingly taken on the role of a kind of “homefluencers” [127], especially with regard to specific health-related issues such as vaccination in general [128]. Thus, the follower base of SMIs could also be used as a target group of electronic word of mouth to increase use of PRWs in the long run. However, special attention should be paid to choosing the most suitable SMI according to the fit between their personality, their follower base, and the specific PRW provider, as it has been widely investigated in commercial realms such as brand relationships [129-131]. Increased use of PRWs could be advantageous not only for PRW providers but also for patients and physicians. Higher PRW use could lead to a higher average number of ratings per physician, which could increase their representativeness [132]. From the physicians’ perspective, PRWs enable them to achieve a positive external impact and learn from feedback and offer them avenues to improve their service quality [133].

### Directions for Future Research

This study investigated not only whether *social influence* exerts an impact on the *behavioral intention* to use PRWs but also how this influence emerges. However, when focusing on this crucial independent variable, several other possible influencing variables could be interesting as well. These include, in line with the UTAUT [25], for example, *effort expectancy*, *facilitating conditions*, *hedonic motivation*, or *habit* [24,26,49,134]. In future investigations, it might be possible to use additional moderator variables (eg, the area where people live [rural vs urban]). To sum up, further elaboration on the influencing chain to explain behavioral intention to use PRWs by including other variables of interest could be an important issue for future research.

## Appendix

Multimedia Appendix 1 Questionnaire for study 1.

Multimedia Appendix 2 Questionnaire for study 2.

Multimedia Appendix 3 Example equation and attention check.

Multimedia Appendix 4 Graphical data cleansing description.

## Abbreviations

PRW: physician rating website
SMI: social media influencer
TAM: technology acceptance model
TIE: theory of information economics
TPB: theory of planned behavior
TRA: theory of reasoned action
UTAUT: Unified Theory of Acceptance and Use of Technology
