# Supplementary material for: The Impact of Social Influence on the Intention to Use Physician Rating Websites: Moderated Mediation Analysis Using a Mixed Methods Approach
Source: J Med Internet Res. 2022 Nov 14;24(11):e37505. doi: 10.2196/37505 (PMC9706386; doi:10.2196/37505)
Supplement: Multimedia Appendix 1 [file jmir_v24i11e37505_app1.pdf]

Multimedia Appendix 1

Variable	Sources	Original measurement	Measurement adapted for research study
**1. Demographic & Psychographic Variables**			
**Gender**	[84]	Gender<br><br>1. Male<br>2. Female	Gender<br><br>1. Male<br>2. Female
**Age**	[84]	Year of Birth:	Year of Birth:
**Education**	[84]	Highest level of education:<br>1. Without school qualification<br>2. Secondary general school<br>3. Polytechnic secondary school<br>4. Intermediate secondary school<br>5. High school diploma / A-levels<br>6. University degree<br>7. Postdoctoral degree / Professor	Highest level of education:<br>1. Without school qualification<br>2. Secondary general school<br>3. Polytechnic secondary school<br>4. Intermediate secondary school<br>5. High school diploma / A-levels<br>6. University degree<br>7. Postdoctoral degree / Professor
**Marital Status**	[84]	Marital status:<br>1. Single<br>2. Close-partnered<br>3. Married<br>4. Divorced<br>5. Widowed<br>6. No answer	Marital status:<br>1. Single<br>2. Close-partnered<br>3. Married<br>4. Divorced<br>5. Widowed<br>6. Other

**Occupation**	[85]	Please indicate your current occupation:<br>1. Self-employed<br>2. Civil servant<br>3. Employee<br>4. Apprentice<br>5. Unemployed<br>6. Pensioner<br>7. Student (pupil)<br>8. Student (Univ./tech. univ.)<br>9. Other<br>10. I do not wish to answer this question	Please indicate your current occupation:<br>1. Self-employed<br>2. Civil servant<br>3. Employee<br>4. Apprentice<br>5. Unemployed<br>6. Pensioner<br>7. Student (pupil)<br>8. Student (Univ./tech. univ.)<br>9. Other
**Area of Living**	[86]	Area of Residence:<br>1. Urban<br>2. Rural	Area of Residence:<br>1. Urban<br>2. Rural
**2. Dependent and Independent Variables**			
**Social Influence**	[24]<br><br>[26]	The scale options ranged from 1 (“strongly disagree”) to 5 (“strongly agree”).<br><br>1. People who influence my behaviour think that I should use the system.<br>2. People who are important to me think that I should use the system.<br>3. People whose opinions that I value prefer that I use mobile Internet.	Experimental Manipulation<br><br>Please imagine that someone, who influences your behavior and/or is important to you and/or whose opinion is appreciated, has recommended the usage of PRWs to you.

**Credibility**	[87]	The scale options ranged from 1 (“strongly disagree”) to 9 (“strongly agree”).<br><br>1. The endorser seems to be credible<br>2. The endorser seems to be reliable<br>3. The endorser seems to be honest<br>4. The endorser seems to be sincere<br>5. The endorser seems to be trustworthy<br>6. The endorser seems to have expert knowledge<br>7. The endorser seems to be experienced<br>8. The endorser seems to disseminate knowledgeable content<br>9. The endorser seems to be qualified<br>10. The endorser seems to be knowledgeable	The scale options range from 1 (“strongly disagree”) to 7 (“strongly agree”).<br><br>1. PRWs seem to be credible<br>2. PRWs seem to be reliable<br>3. PRWs seem to be honest<br>4. PRWs seem to be sincere<br>5. PRWs seem to be trustworthy<br>6. PRWs seem to have expert knowledge<br>7. PRWs seem to be experienced<br>8. PRWs seem to contain knowledgeable content<br>9. PRWs seem to be qualified<br>10. PRWs seem to be knowledgeable
**Performance Expectancy**	[49]<br><br>[88]	The scale options ranged from 1 (“strongly disagree”) to 5 (“strongly agree”).<br><br>1. I think that the portal is a useful tool<br>2. By using the portal I feel I have more control over my health<br>3. Using EHR Portals will enhance my effectiveness in managing my healthcare.<br>4. Overall, EHR Portals will be useful in managing my healthcare	The scale options range from 1 (“strongly disagree”) to 7 (“strongly agree”).<br><br>1. I think that PRWs are a useful tool<br>2. By using PRWs I feel I have more control over my health<br>3. Using PRWs will enhance my effectiveness in managing my healthcare
**Behavioral Intention to Use PRWs**	[26]	The scale options ranged from 1 (“strongly disagree”) to 5 (“strongly agree”).<br><br>1. I intend to continue using mobile Internet in the future<br>2. I will always try to use mobile Internet in my daily life<br>3. I plan to continue to use mobile Internet	The scale options range from 1 (“strongly disagree”) to 7 (“strongly agree”).<br><br>1. I intend to use PRWs in the future<br><br>2. I will try to use PRWs<br><br>3. I plan to use PRWs
