# Supplementary material for: The Impact of Social Influence on the Intention to Use Physician Rating Websites: Moderated Mediation Analysis Using a Mixed Methods Approach
Source: J Med Internet Res. 2022 Nov 14;24(11):e37505. doi: 10.2196/37505 (PMC9706386; doi:10.2196/37505)
Supplement: Multimedia Appendix 3 [file jmir_v24i11e37505_app3.pdf]

Multimedia Appendix 3

Equation Example:

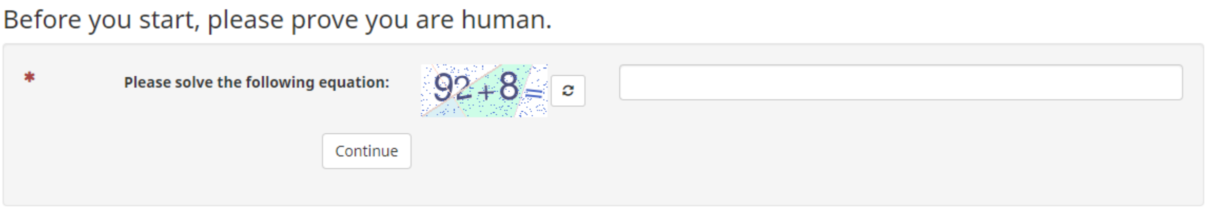

Attention Check:

Physician Rating Websites offer healthcare consumers the opportunity to anonymously rate their doctor. These ratings could help future or potential patients in the decision-making process regarding their future medical care. To continue our research focusing on physician rating portals, we would like to learn more about you and your interaction with those online platforms. As a first step, we are interested in whether you take the time to read the text content comprehensively. To confirm that you have read the instructions, please ignore the next question and select only the option 'Other'.

<u>Which state do you come from?</u>

- ○ **Burgenland**
- ○ **Carinthia**
- ○ **Lower Austria**
- ○ **Upper Austria**
- ○ **Salzburg**
- ○ **Styria**
- ○ **Tyrol**
- ○ **Vorarlberg**
- ○ **Vienna**
- ○ **Other**
