# Supplementary figures and images for: The Impact of Social Influence on the Intention to Use Physician Rating Websites: Moderated Mediation Analysis Using a Mixed Methods Approach
Source: J Med Internet Res. 2022 Nov 14;24(11):e37505. doi: 10.2196/37505 (PMC9706386; doi:10.2196/37505)

*Multimedia Appendix 4*

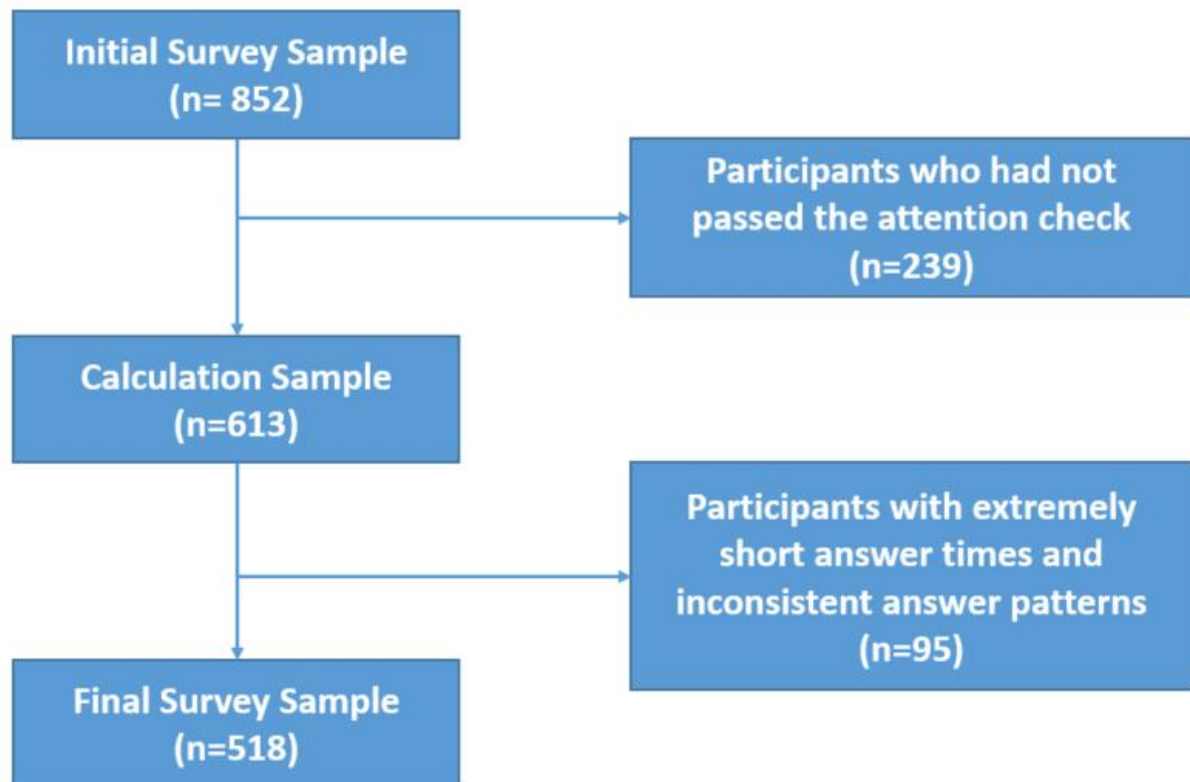

Supplement: Multimedia Appendix 4 [file jmir_v24i11e37505_app4.pdf]
